# All-in-One High-Power-Density Vibrational Energy Harvester with Impact-Induced Frequency Broadening Mechanisms

**DOI:** 10.3390/mi12091083

**Published:** 2021-09-08

**Authors:** Yongqi Cao, Weihe Shen, Fangzhi Li, Huan Qi, Jiaxiang Wang, Jianren Mao, Yang Yang, Kai Tao

**Affiliations:** 1 Ministry of Education Key Laboratory of Micro and Nano Systems for Aerospace, Northwestern Polytechnical University, Xi’an 710072, China; caoyq@mail.nwpu.edu.cn (Y.C.); 2017300086@mail.nwpu.edu.cn (F.L.); jiaxiang@mail.nwpu.edu.cn (J.W.); 2 Research & Development Institute in Shenzhen, Northwestern Polytechnical University, Shenzhen 518057, China; yang_yang@cqu.edu.cn; 3 Science and Technology on Space Physics Laboratory, China Academy of Launch Vehicle Technology, Beijing 100076, China; weihenihao@gmail.com (W.S.); maoren_99@163.com (J.M.); 4 Beijing Institute of Astronautical Systems Engineering, China Academy of Launch Vehicle Technology, Beijing 100076, China; qi_huan@sina.com

**Keywords:** vibration energy harvesting, all-in-one, frequency broadening

## Abstract

This paper proposes an electrostatic-piezoelectric-electromagnetic hybrid vibrational power generator with different frequency broadening schemes. Both the nonlinear frequency broadening mechanisms and the synergized effect of the electrostatic-piezoelectric-electromagnetic hybrid structures are investigated. The structure and performance of the composite generator are optimized to improve the response bandwidth and performance. We propose that the electrostatic power generation module and the electromagnetic power generation module be introduced into the cantilever beam to make the multifunctional cantilever beam, realizing small integrated output loss, high output voltage, and high current characteristics. When the external load of the electrostatic power generation module is 10 kΩ, its peak power can reach 3.6 mW; when the external load of the piezoelectric power generation module is 2 kΩ, its peak power is 2.2 mW; and when the external load of the electromagnetic power generation module is 170 Ω, its peak power is 0.735 mW. This means that under the same space utilization, the performance is improved by 90%. Moreover, an energy management circuit (ECM) at the rear end of the device is added, through the energy conditioning circuit, the device can directly export a 3.3 V DC voltage to supply power to most of the sensing equipment. In this paper, the hybrid generator’s structure and performance are optimized, and the response bandwidth and performance are improved. In general, the primary advantages of the device in this paper are its larger bandwidth and enhanced performance.

## 1. Introduction

Over the past few decades, tremendous advances have been made in microelectronic systems, with devices becoming smaller and requiring less energy [1]. However, limited by the service life and energy density of the traditional batteries, the power supply scheme of these systems is still a challenge. Therefore, researchers hope to design some devices to harvest the energy in the environment to power these microelectronic systems autonomously. Meanwhile, these low-power electronic devices pose a challenge to cheap, flexible, portable, and sustainable energy resources [2,3,4,5,6,7,8,9]. Therefore, many researchers are devoted to the demand of these microelectronic systems, among which vibration energy harvesting technology has become a research hotspot [10,11,12,13,14,15,16,17,18,19,20,21].

The energy conversion mechanism based on ambient vibration is electrostatic (ES), electromagnetic (EM), piezoelectric (PE), and triboelectric [22,23,24,25,26,27]. In terms of the electrostatic mechanism, Naruse et al. [28] present a low-frequency electrostatic micro-vibrational energy harvester (VEH) supported by microspheres. The result shows that the output power can reach 40 μW under the excitation of 2 Hz and 0.4 g vibration. Nobuhide Kasagi et al. [29] present an microelectromechanical systems (MEMS) electret generator with nonlinear spring. Additionally, the power output of 1 µW has been obtained at 63 Hz. Ugur Erturun et al. [30] present a vibration energy harvester using push–pull electrostatic conversion. The stored energy of ~900 µJ and the power of ~15 µW are obtained by charging a capacitor for around a minute. Additionally, the output voltage can reach 318 V. James E. West et al. [31] demonstrate an energy harvester that combines a piezoelectric nanogenerator and an electret-based electrostatic generator. The maximum peak–peak voltage output of the electrostatic part is 500 V at 20 Hz. The short-circuit current output of the electrostatic part is 1.1 μA at 10 Hz. In these studies, the electrostatic energy harvesting method can output a large voltage, but the current generated is small.

In terms of the electromagnetic mechanism, Kulkarni et al. [32] present a miniature electromagnetic VEH with the help of MEMS technology. By changing the structure, the maximum output voltage and power of the device can reach 950 mV and 85 μW. Külah et al. [33] present an electromagnetic VEH that converts a low-frequency vibration into a higher-frequency vibration through frequency conversion technology. The maximum output voltage and power obtained by VEH at a natural frequency of 64 Hz are 6 mV and 120 nW, respectively. Peihong Wang [34] uses MEMS technology to make a new electromagnetic VEH. When the acceleration is 0.8 g and the frequency is 280.9 Hz, the maximum output voltage and power are 101 mV and 19.5 μW, respectively. Kankana Paul et al. [35] present the design and performance of fully integrated EM vibration energy harvesters on the scale of microelectromechanical systems (MEMS). The device produces a power density as high as 52 µW/cm^3^ at 1 g acceleration. The nonlinear counterpart enhances the bandwidth almost six times to 25 Hz at the cost of reduced power density. In these studies, the voltage performance of the small-size electromagnetic energy generator in the low-frequency environment is not very prominent, but the current generated using the electromagnetic energy harvesting method is large.

Additionally, in terms of the piezoelectric mechanism, a small piezoelectric cantilever beam type linear narrow-band generator is proposed by Bai et al. [36], which can acquire the average power of 50 and 20 μW, respectively, when placed on the arm and top of the human body, and the power density is 0.35 and 0.14 μW/mm^3^, respectively. However, linear narrow-band generators cannot adapt to variable environmental vibrations at any time and cannot provide a stable output. Therefore, Shahruz et al. [37] proposed an array piezoelectric broadband generator. The different natural frequencies of the array cantilever beams increase the bandwidth of the generator. Xue et al. [38] also proposed a piezoelectric generator with a bimorph cantilever beam array, which uses different resonance frequencies caused by different wafer thicknesses to increase the bandwidth of the generator. However, the average power of the array generator is low. Leland et al. [39] proposed a clamped beam adjustable resonant generator with axial compression preload. This generator can change the beam stiffness to change the natural frequency and increase the bandwidth. However, the adjustable resonant generator cannot respond quickly to changes in excitation. In addition to the above two types of generators that achieve broadband, Li et al. [40] proposed a hardened extrusion-type nonlinear generator that can harvest energy at a lower vibration level. Alireza Rezaniakolaie et al. [41] proposed a trapezoid harvesting multi-beam structure with macro-fiber-composite toward the energy conversion enhancement of piezoelectric energy harvesters from wideband excitation signals. The power generation by this harvester is 84% greater than a common multi-beam design at a 47%-reduced volume, resulting in a 160% power density improvement. Additionally, Weiqun Liu et al. [42] proposed a nonlinear generator with curved surface fixtures, effectively increasing the bandwidth and power.

In general, there are many methods to implement broadband generators, but the nonlinear generator is a more effective solution due to its large bandwidth when carrying out energy harvesting. Therefore, this paper proposes a hybrid nonlinear generator with vibration energy harvesting technologies. On the one hand, we adopt the curved fixture structure to increase the bandwidth. On the other hand, the electrostatic [43,44,45,46,47,48] generator module and the electromagnetic module are integrated with the piezoelectric generator module to improve the output power of the nonlinear generator. We propose that the electrostatic power generation module and the electromagnetic power generation module be introduced into the cantilever beam to make the multifunctional cantilever beam, realizing small integrated output loss, high output voltage, and high current characteristics.

## 2. The Conception of Device

**Figure 1:**
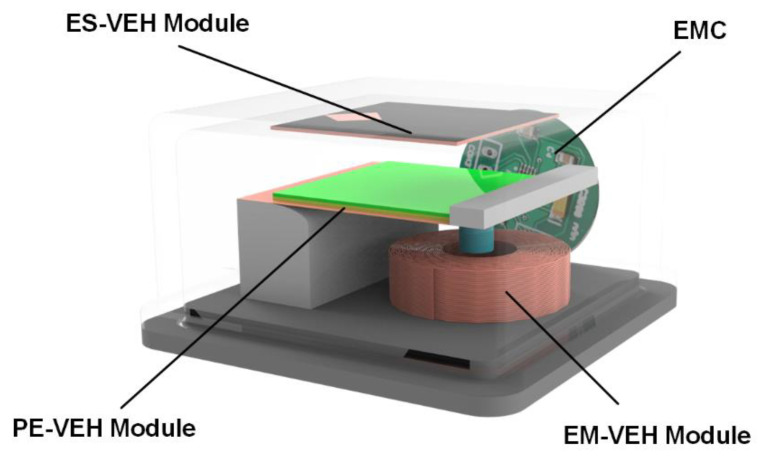
Overall diagram of the electrostatic-piezoelectric-electromagnetic hybrid vibrational power generator: ES-VEH module is the electrostatic power generation module, PE-VEH module is the piezoelectric power generation module, EM-VEH module is the electromagnetic power generation module, and EMC is the energy management circuit module.

This paper designs a hybrid nonlinear generator that combines three power generation methods. The structure design of the power generation is a multifunctional cantilever beam structure composed of the piezoelectric power generation module, a partial structure of the electrostatic power generation module, and a partial structure of the electromagnetic power generation module. As shown in Figure 1, this paper integrates the piezoelectric power generation module, the electrostatic power generation module, the electromagnetic power generation module, and the energy conditioning circuit module based on the LTC3588 chip into a package.

**Figure 2:**
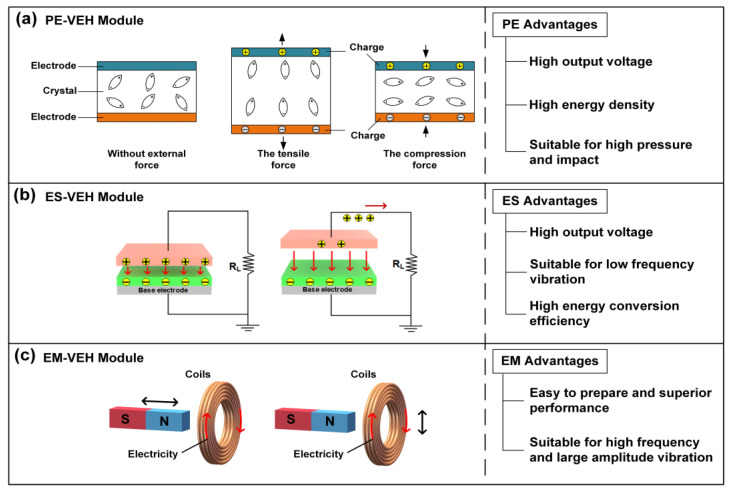
The principle and advantages of the three power generation modules.

The power generation principle of the three power generation modules is shown in Figure 2. The piezoelectric power generation module in this paper is mainly based on the positive piezoelectric effect of piezoelectric materials. Its working principle is as shown in Figure 2a, if the pressure to the direction of the polarization is applied to the piezoelectric chip, due to the compression and deformation of the piezoelectric chip, the distance between the positive and negative charges in the piezoelectric layer will decrease and the polarization strength of the chip will be weakened, which causes a part of the free electrons on the surface electrode of the piezoelectric layer to be released and a discharge phenomenon occurs. After the pressure is removed, the distance between the positive and negative charges in the chip will increase, and the polarization strength will also increase. At this time, a part of the free charges will be adsorbed on the surface of the upper and lower electrodes of the piezoelectric layer and a charging phenomenon will occur.

In the actual working process, the device’s piezoelectric layer constantly repeats the charging and discharging process under the influence of the alternating forces provided by the external vibration environment in order to realize the conversion of mechanical energy into electrical energy. The working principle of the electrostatic power generation module is to convert external mechanical disturbances into capacitance changes of variable capacitors under a constant bias voltage, which causes the charge flow between the two electrodes, thereby converting mechanical energy into electrical energy under external excitation. The FEP is a dielectric with permanent charge or dipole polarization. It can form a permanent electric field around it; therefore, it can be used as a constant voltage source to provide a bias voltage for the electrostatic power generation module.

The working principle of the electrostatic power generation module based on out-of-plane motion is shown in Figure 2b. Due to the electrostatic induction, the negative charge in the electret material on the movable electrode will generate the corresponding positive charge on the fixed electrode, and the positive charge will flow back and forth with external disturbances, thereby converting the mechanical energy into electric energy.

The working principle of the electromagnetic power generation module in this paper is based on the electromagnetic induction, which converts the change of the magnetic flux in the coil into the induced current. It is generally composed of a permanent magnet, a coil, and an elastic unit. When the outside vibrates, the elastic unit will induce the vibration of the outside, causing relative movement between the magnet and the coil, and the coil will sense the changing magnetic flux to generate the output of the electrical energy. The relative movement between the permanent magnet and the coil can be either the permanent magnet moving without the coil moving, or the coil moving without the permanent magnet moving (Figure 2c). The electromagnetic power generation module of this paper uses the electromagnetic power generation method that the permanent magnet moves, and the coil does not move.

Among them, the piezoelectric power generation module uses an MEMS piezoelectric vibration energy harvester design scheme. The dual-crystal cantilever beam of this module uses a metal substrate piezoelectric film multilayer composite structure preparation process, which mainly includes the surface polishing of the material to be bonded, the hot-press bonding of conductive silver glue, the mechanical thinning of the piezoelectric layer, metal electrode sputtering, laser cutting, fixing of the cantilever beam, and the lead process. To enhance the electrical performance, the piezoelectric power generation module adopts the bonding and thinning technology based on the intermediate layer to fabricate the film with excellent piezoelectric properties. At the same time, the mass block is attached to the free end of the cantilever beam, thus, further improving the output performance.

In the piezoelectric power generation module, the PZT piezoelectric material is used as the piezoelectric functional layer. The piezoelectric cantilever beam is used as the main structure of the energy harvester, and a mass block is added at the free end of the beam. The electrostatic power generation module is composed of a movable electrode and a fixed electrode. The fixed electrode is a Cu material layer attached to the top of the internal side of the package shell. The movable electrode is the pre-charged electret material film (FEP) attached to the Cu material layer and bonded to the piezoelectric ceramic generating layer. The electromagnetic power generation module is composed of an inductance coil and a cylindrical permanent magnet. The inductance coil is placed in the center of the package shell base, and the permanent magnet is glued under the cantilever beam to provide a magnetic field for the electromagnetic power generation module.

**Figure 3:**
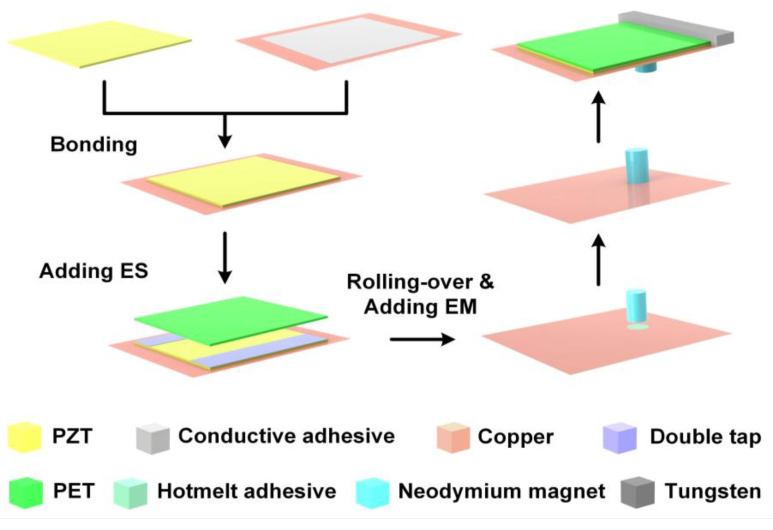
The manufacturing process of the multifunctional cantilever beam.

In this way, the piezoelectric power generation module, the electret layer of the electrostatic power generation module, and the permanent magnet of the electromagnetic power generation module are integrated into a multifunctional cantilever beam that incorporates three power generation methods. The manufacturing process of the multifunctional cantilever beam is shown in Figure 3.

The energy conditioning circuit mainly consists of LTC3588-1, which integrates a full-wave bridge rectifier with low loss and a buck converter with high efficiency. The package is a 3D printed shell made of resin material. The entire device collects vibration energy in the environment by piezoelectric and electrostatic power generations. It then converts into a well-regulated output to power application microcontrollers, sensors, data converters, and wireless transmission components.

**Figure 4:**
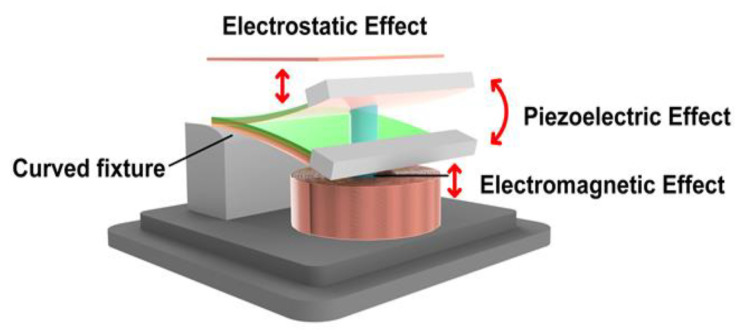
The movement of the three power generation modules.

Figure 4 shows the movement of the three power generation modules when the device is subjected to vibration. When affected by the external vibration, the free end mass of the multifunctional cantilever beam vibrates the cantilever beam up and down. The piezoelectric crystal produces the piezoelectric effect. The distance between the FEP movable electrode layer on the piezoelectric layer and the fixed electrode on the top of the shell changes, and thus the electrostatic effect is generated. The permanent magnet at the bottom of the cantilever beam starts to move up and down in the inner ring of the inductor coil to generate electromagnetic induction to complete the power generation process. At the same time, this article explores the nonlinear effect, and uses the bending fixture to broaden the frequency band. The advantages of the bending fixture will be given in the experimental section.

The vibration of the multifunctional cantilever beam causes the piezoelectric, electrostatic, and electromagnetic power generation modules to start working at the same time. The power generation of the three power generation modules is based on the movement of the multifunctional cantilever beam, which simplifies the complexity of the device.

## 3. Experimental Design

Firstly, this paper explores the nonlinear effect and designs two fixtures; one is an ordinary linear fixture, the other is a curved fixture. The design will test the respective characteristics of the two fixtures in an experiment for comparative analysis. First of all, based on these two fixtures, stoppers will be added at the middle and the end of the ordinary linear fixtures to explore the nonlinear effects of piezoelectric cantilever beams under three different structures. When the cantilever beam is deformed by external excitation, the cantilever beam will come into contact with the fixture, the effective length of the beam will be shortened, and the rigidity of the system will increase, thereby introducing nonlinear effects.

**Figure 5:**
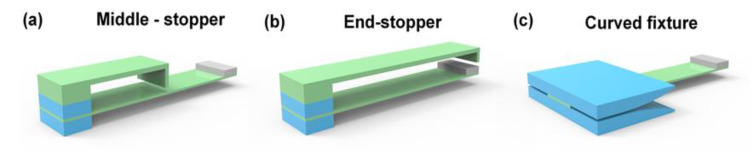
The mechanical structure of three non-linear test structures.

As shown in Figure 5, it shows the mechanical structure of the cantilever beam with stoppers at the middle and end of the cantilever on the basis of ordinary linear clamps and the use of curved clamps. The two cases of the ordinary linear fixture setting stoppers are shown in Figure 5a,b. The stoppers are, respectively, set at the middle and the end of the cantilever beam. When the cantilever beam is deformed due to vibration, the cantilever beam contacts the stopper; at this time, the effective length of the cantilever beam will change greatly, but this change is fixed. Figure 5c shows a curved fixture, which makes the effective length of the cantilever beam have different changes in one working cycle. Therefore, the nonlinear effect curves of the three structures are also significantly different.

Additionally, this paper has carried on a certain theoretical derivation. In the general linear piezoelectric vibration power generation theoretical model, $M_{eq}$ is the equivalent mass of the piezoelectric power generation module, ξ is the damping coefficient, $K_{eq}$ is the equivalent stiffness of the piezoelectric power generation module, u(t) is the vibration of the cantilever piezoelectric vibrator displacement, ∂ is the electromechanical coupling coefficient of the piezoelectric power generation module, and $R_{L}$ is a purely resistive load. When the external excitation F(t) affects, the dynamic equation of the equivalent mass block can be obtained according to Newton’s second law, and the lumped parameter equivalent model of the piezoelectric power generation module shown in the following Equation (1) can be obtained according to Kirchhoff’s law:

(1) $$\left\{ \begin{array}{l} M_{eq}\ddot{u}(t)+\xi \dot{u}(t)+K_{eq}u(t)+\partial V_{0}(t)=F(t)\\ \partial \dot{u}(t)=C_{p}{\dot{V}}_{0}(t)+V_{0}(t)/R_{L} \end{array}\right.$$

where $V_{0}\left(t\right)$ is the output voltage of the piezoelectric power generation module, and $\partial V_{0}\left(t\right)$ reflects the reaction force of inverse piezoelectric effect on the cantilever piezoelectric vibrator.

In the three fixtures shown in Figure 5, a theoretical model is established for two structures of the nonlinear piezoelectric power generation module with a stopper in Figure 5a,b. The damping coefficient, equivalent stiffness, and electromechanical coupling coefficient are expressed as a function of vibration displacement ξ(u), $K_{eq}\left(u\right)$, and Θ(u), in order to obtain the general form of the system model of the nonlinear piezoelectric power generation module, as shown in the following Equation (2):

(2) $$\left\{ \begin{array}{l} M_{eq}\ddot{u}(t)+\xi (u)\dot{u}(t)+K_{eq}(u)u(t)+\Theta (u)Q(t)/C_{p}=F(t)\\ \Theta (u)u(t)=C_{p}Q(t)R_{L}+Q(t)\\ \xi (u)=a_{1}+a_{2}\left|\dot{u}\right|\\ K_{eq}=b_{1}+b_{2}u^{2}\\ \Theta (u)=d_{1}+d_{2}\sqrt{u} \end{array}\right.$$

where Q(t) is the charge generated at both ends of the electrode; ξ(u) is the square damping, where $\alpha_{1}$ is the linear damping coefficient, and $\alpha_{2}$ is the nonlinear damping coefficient; $K_{eq}\left(u\right)$ is the nonlinear stiffness, where $b_{1}$ is the linear stiffness coefficient, $b_{2}$ is the nonlinear stiffness coefficient; and Θ(u) is nonlinear electromechanical coupling coefficient, where $d_{1}$ is the linear electromechanical coupling coefficient, and $d_{2}$ is the nonlinear electromechanical coupling coefficient.

After integration and dimensionless processing, the vibration displacement, output charge, and time are standardized through the following Equation (3), and the theoretical general model of the nonlinear piezoelectric power generation module is obtained, as shown in Equation (4):

(3) $$\left\{ \begin{array}{l} u(t)=c_{z}z(\tau )\\ Q(t)=c_{q}q(\tau )\\ t=\tau \sqrt{m/b_{1}} \end{array}\right.$$

where $c_{z}$ and $c_{q}$ are standardization coefficients in units of m and C, respectively. τ is the standardization time.

(4) $$\left\{ \begin{array}{l} \ddot{z}+(\frac{a_{1}}{\sqrt{mb_{1}}}\dot{z}+\frac{c_{z}a_{2}}{m}\left|\dot{z}\right|\dot{z})+(z+\frac{b_{2}{c_{z}}^{2}}{b1}z^{3})+\frac{c_{q}}{b_{1}c_{z}C_{p}}(d_{1}+d_{2}\sqrt{c_{z}\left|z\right|})q\\ =F(\tau \sqrt{m/b_{1}})/(b_{1}c_{z})\\ C_{p}R_{L}\dot{q}\sqrt{\frac{b_{1}}{m}}-\frac{c_{z}}{c_{q}}(d_{1}+d_{2}\sqrt{c_{z}\left|z\right|})z+q=0 \end{array}\right.$$

Simplified:

(5) $$\left\{ \begin{array}{l} \ddot{z}+(2\mu \dot{z}+\eta \left|\dot{z}\right|\dot{z})+(z+\varphi z^{3})+\varepsilon (\theta +\beta \sqrt{\left|z\right|})q\\ =F(\tau \sqrt{m/b_{1}})/(b_{1}c_{z})\\ \rho \dot{q}-(\theta +\beta \sqrt{\left|z\right|})z+q=0 \end{array}\right.$$

Making $2\mu =\alpha_{1}/\sqrt{mb_{1}}$, $\eta =c_{z}\alpha_{2}/m$, $\varphi =b_{2}c_{z}^{2}/b_{1}$, $\varepsilon =c_{q}^{2}/\left(b_{1}c_{z}^{2}C_{p}\right)$, $\theta =d_{1}c_{z}/c_{q}$, $\beta =d_{2}c_{z}\sqrt{c_{z}}/c_{q}$, and $\rho =C_{p}R_{L}\sqrt{b_{1}/m}$. z is the dimensionless vibration displacement, μ is the dimensionless linear damping coefficient, η is the dimensionless nonlinear damping coefficient, φ is the dimensionless nonlinear stiffness coefficient, θ is the dimensionless linear electromechanical coupling coefficient, β is the dimensionless nonlinear electromechanical coupling coefficient, q is the dimensionless output charge, and ρ is the dimensionless pure resistance load.

When the input excitation is $F\left(t\right)=F_{0}sin\left(\Omega^{\prime }t\right)$, the normalized formula on the right side of Equation (5) is as follows:

(6) $$\hat{F}(\tau )=F_{0}/(b_{1}c_{z})sin(\Omega^{\prime }\sqrt{m/b_{1}\tau })=\gamma sin(\omega \tau )$$

where $\gamma =F_{0}/\left(b_{1}c_{z}\right)$ and $\omega =\sqrt{m/b_{1}}\Omega^{\prime }$. Equation (5) is further transformed into the following:

(7) $$\left\{ \begin{array}{l} \ddot{z}+(2\mu \dot{z}+\eta \left|\dot{z}\right|\dot{z})+(z+\varphi z^{3})+\varepsilon (\theta +\beta \sqrt{\left|z\right|})q\\ =\gamma sin(\omega \tau )\\ \rho \dot{q}-(\theta +\beta \sqrt{\left|z\right|})z+q=0 \end{array}\right.$$

Then, the average power in a period *T* is as follows:

(8) $${\tilde{P}}_{avg}=\frac{1}{T}{\int_{0}^{T}\rho \dot{q}(\tau )}^{2}d\tau$$

which, when converted to dimensional average power, is as follows:

(9) $$P_{avg}=\frac{c_{q}^{2}}{C_{q}}\sqrt{\frac{b_{1}}{m}}{\tilde{P}}_{avg}$$

Figure 5c shows the designed nonlinear piezoelectric power generation module of the bending fixture. The elastic restoring force $F_{r}=K_{eq}\left(u\right)u\left(t\right)$ is added to the established theoretical general model of the nonlinear piezoelectric power generation module to obtain the theoretical model of the nonlinear piezoelectric power generation module of the bending fixture, as shown in Equation (6).

(10) $$\left\{ \begin{array}{l} M_{eq}\ddot{u}(t)+\xi \dot{u}(t)+F_{r}+\partial V_{0}(t)=F(t)\\ \partial \dot{u}(t)=R_{L}C_{p}{\dot{V}}_{0}(t)+V_{0}(t)\\ F_{r}=f_{1}u(t)+f_{2}u{\left(t\right)}^{3}\\ k=dF/du(t)=f_{1}+3f_{2}u{\left(t\right)}^{2} \end{array}\right.$$

where k is the nonlinear stiffness, where $f_{1}$ is the linear stiffness coefficient, and $f_{2}$ is the nonlinear stiffness coefficient.

Secondly, this paper also designs the experiment of the coupling characteristics among the three power generation modules, and explores the respective power generation characteristics of the three power generation modules and discusses the effect that the coupling can achieve.

## 4. Testing Results

**Figure 6:**
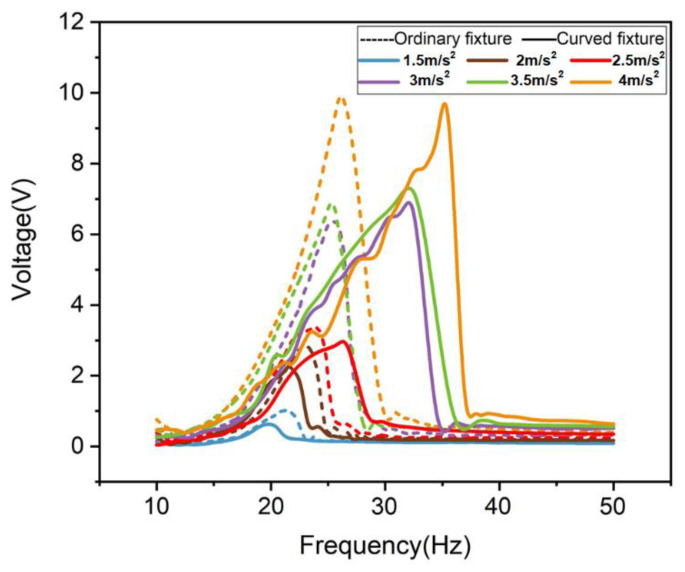
Ordinary fixture and curved fixture test comparison.

Firstly, this paper explores the influence of the ordinary linear fixture and the curved fixture on the performance of piezoelectric power generation structures. As shown in Figure 6, when the external load is 10 kΩ and the excitation amplitude increases from 1.5 to 4 m/s^2^, the non-linear effect exhibited by the curved fixture makes the frequency band continue to expand, which shows the good non-linear effect of the curved fixture. It can be seen that the bandwidth of the curved fixture is 25 to 33 Hz under 3 m/s^2^, while the bandwidth of the ordinary linear fixture is 22 to 27 Hz. The curved fixture bandwidth under 3.5 m/s^2^ is 26 to 35 Hz, and the bandwidth of the ordinary linear fixture is 22 to 27 Hz. At 3 and 3.5 m/s^2^, the curved fixture has an expanded bandwidth compared to the ordinary linear fixture. Compared with the ordinary linear fixture, the curved fixture’s bandwidth increased by 60 and 80%, respectively. At this time, the power of the curved fixture is 112 and 120% of the ordinary linear fixture.

**Figure 7:**
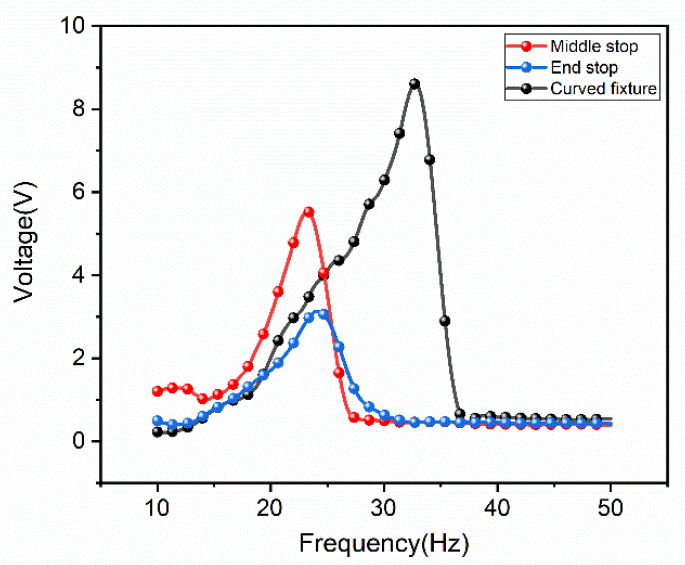
Three structural non-linear tests.

Secondly, based on the ordinary linear fixture, the experiment set stoppers at the middle and the end of the cantilever, and the nonlinear effects of the cantilever caused by these two structures and using the curved fixture are tested in Figure 7. It can be seen in the figure that although the non-linearity of placing the stopper at the end may not be obvious due to the small acceleration, it can be seen that the nonlinear effect of placing the stopper at the middle and using the curved fixture is more obvious. Obviously, the bandwidth and performance of using the curved fixture have been greatly improved compared to the ordinary linear fixture of setting stoppers. Using the curved fixture, the performance of the device is improved by nearly two times.

**Figure 8:**
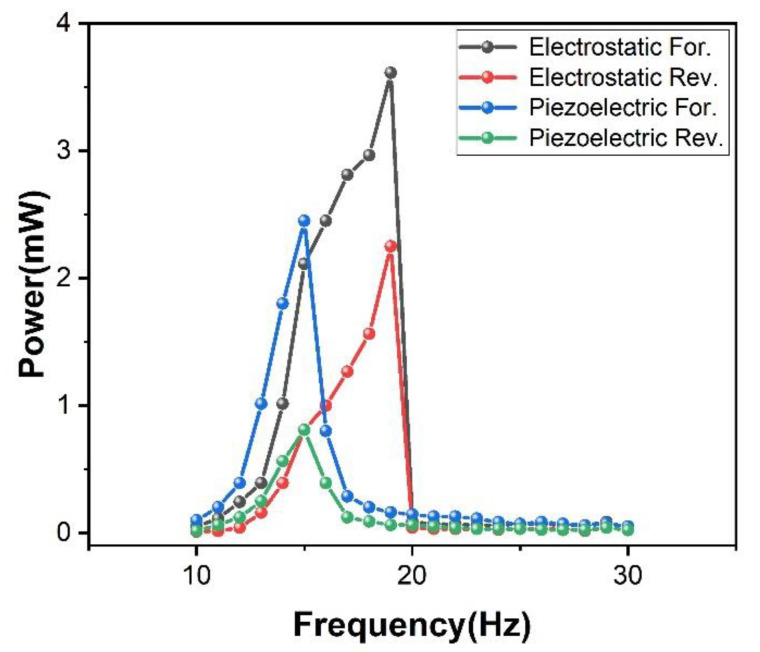
Non-linear curve with electrostatic structure added. For. is forward sweep and Rev. is reverse sweep.

After exploring the advantages of the curved fixture, the experiment adds the electrostatic power generation module on this basis. The result is shown in Figure 8. After adding the electrostatic power generation module, because of the structure design of the electrostatic power generation module, it also shows a nonlinear effect. The half-power bandwidth of the piezoelectric cantilever beam is 14 to 19 Hz, and the half-power bandwidth of the electrostatic power generation module is 14 to 19.5 Hz. At this time, the electrostatic external load is 10 kΩ and the peak power is 3.6 mW, while the piezoelectric external load is 2 kΩ and the peak power is 2.2 mW. It can be seen that although the addition of the electrostatic power generation module does not expand the frequency bandwidth, it improves the performance of the generator. This means that under the same space utilization, the performance is improved by 60%.

**Figure 9:**
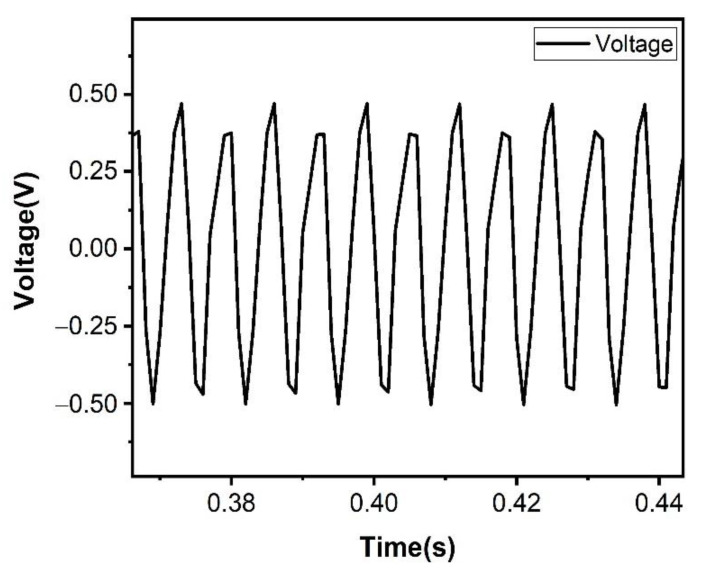
The voltage test of the electromagnetic power generation module.

In addition, this paper also explores the specific performance of the electromagnetic power generation module. After testing, the contribution of the electromagnetic module is 0.5 V of the voltage. The voltage test is shown in Figure 9. Although the electromagnetic power generation module has no nonlinear effect and does not broaden the frequency band of the device, its high current characteristics can improve the output current of the device.

**Figure 10:**
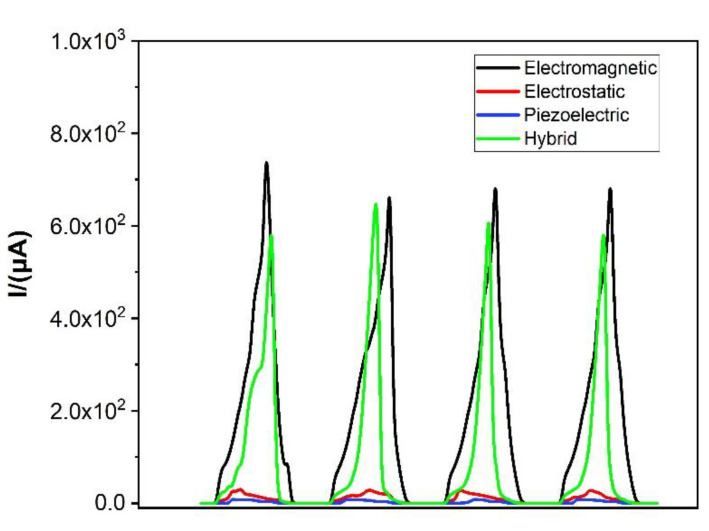
Three kinds of module current comparison and hybrid current.

To explore the influence of the high current characteristics of the electromagnetic module on the output current of the device, a design experiment is designed to test the current of the three modules and the current of the entire device. As shown in Figure 10, the electromagnetic high-current characteristics are well preserved, which greatly improves the current output of the device. The electrostatic module current can reach 28 μA, the piezoelectric module current can reach 7 μA, and the electromagnetic module current can reach 764 μA. Although the hybrid current of the three modules has a loss, it can still be seen that the electromagnetic module has increased the device current. The hybrid current can reach 653 μA.

**Figure 11:**
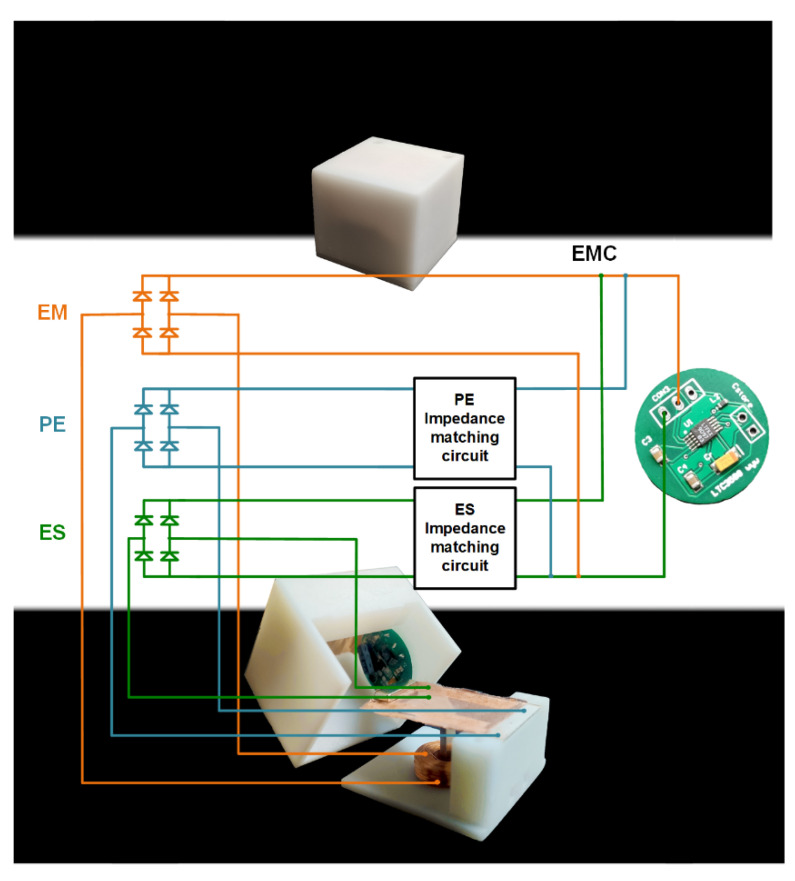
The physical map of the device and the energy conditioning circuit.

In this paper, three kinds of power generation modules are integrated and encapsulated in the 3D printing shell together with the energy conditioning circuit. Figure 11 shows the appearance of the device after packaging and the energy management strategy used in this paper. In the paper, the impedance of PE is 2 kΩ, the impedance of ES is 10 kΩ, and the impedance of EM is only 170 Ω; the electrical impedance of the EM is significantly different from the PE and ES part. To overcome the problem, an additional impedance matching circuit needs to be incorporated in PE and ES after the AC-DC conversion, respectively. The impedance matching circuit is capable of adjusting the impedance of PE and ES to the same level of EM. After that, the DC signals from three mechanisms are superposed together for an improved performance.

This paper integrates the three power generation modules and the energy conditioning circuit in a small-sized package shell. Under ensuring its good performance, the size of the device is reduced as much as possible so that the device can adapt to more application scenes. According to the calculation, the circuit power consumption of the current system is calculated up to 3 mW, and the overall converted energy before the power conditioning circuit is about 6.5 mW. The conversion efficiency of mechanical energy to electrical energy ratio is calculated as 0.12/1.6 = 0.075, 7.5%.

Three kinds of power generation modules are effectively integrated into the confined space. During the working process of the device, the electromagnetic power generation module actively participates in the contribution in the low-frequency domain, and its high current characteristic is fully utilized. In the high-frequency domain, the electrostatic power generation module and the piezoelectric power generation module actively participate in the contribution, and the high-voltage characteristics are fully exerted. Therefore, the device has a wider working frequency domain and achieves a higher energy conversion efficiency in multiple vibration frequency bands. In the same time domain, the multifunctional cantilever beam structure means that the output of the piezoelectric, electrostatic, and electromagnetic power generation modules is basically in the same phase. The integrated output loss is small, and the characteristics of high voltage and high current are realized.

**Figure 12:**
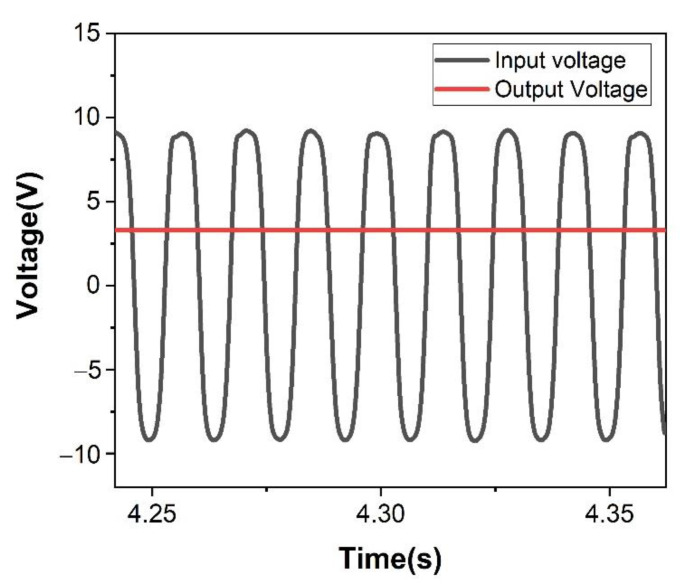
The conversion effect of the energy conditioning circuit.

Finally, there is an energy conditioning circuit at the rear end of the device. The output of the three power generation modules is adjusted by the energy conditioning circuit and the AC is converted into the DC that most sensors can directly use. The conversion effect is shown in Figure 12. After the conditioning of the energy conditioning circuit, it can finally output a 3.3 V DC voltage.

## 5. Conclusions

This paper proposes a hybrid nonlinear generator with the following three power generation modules: piezoelectric, electrostatic, and electromagnetic. The influence of the ordinary linear fixture and the curved fixture on the performance of the generator has been explored. It has been verified that compared with the ordinary linear fixture, the curved fixture has excellent bandwidth expansion capabilities and can improve the performance by 25% compared with the ordinary stopper structure. While exploring the nonlinear effect of the piezoelectric cantilever beam, we have integrated the electrostatic power generation module and the electromagnetic power generation module into the piezoelectric power generation module. After adding the electrostatic power generation module, the half-power bandwidth of the piezoelectric cantilever beam has been increased from 16 to 20 Hz. The peak power of 3.6 mW has been achieved. The half-power bandwidth of the electrostatic structure has been increased from 14.5 to 19.5 Hz, and the peak power is 2.2 mW. Although the addition of electrostatic energy generation module does not expand the frequency bandwidth, it improves the performance of the generator. This means that under the same space utilization, the performance is improved by 60%. In addition, the electromagnetic energy generation module is introduced into the cantilever beam structure, which also contributes to the device. Finally, we have optimized the structure and performance of the hybrid generator and have achieved the improvement of the bandwidth and performance. Moreover, an energy conditioning circuit at the rear end of the device is added. Through the energy conditioning circuit, the device can directly output a DC voltage to supply power to most of the sensing equipment.

In general, the device designed in this paper has the advantages of a larger bandwidth, better performance, and smaller size, and can be used in many vibration environments in limited spaces. Additionally, the device can overcome the shortcomings of the single energy harvesting device, such as the low current of piezoelectric and electrostatic power generation, and the low voltage of electromagnetic power generation, etc., the output performance is better than the single energy harvesting device. Although the hybrid energy harvesting device has these advantages, it also has some problems, such as difficulty in coupling multiple power generation units, large size, and high cost. Therefore, further improving the coupling performance of multiple units of the hybrid energy harvesting device, designing new structures to improve integration and reduce the device size, and reducing costs are things that need to be considered.

## Data Availability

The data that support the findings of this study are available from the first author upon reasonable request.

## References

[1] Pirisi A., Mussetta M., Gruosso G., Zich R.E. Bio-inspired optimization techniques for wireless energy transfer. Proceedings of the International Conference on Electromagnetics in Advanced Applications (ICEAA).

[2] Liu H.C., Zhong J.W., Lee C.K., Lee S.-W., Lin L.W. (2018). A comprehensive review on piezoelectric energy harvesting technology: Materials, mechanisms, and applications. Appl. Phys. Rev..

[3] Li Y.J., Tao K., George B., Tan Z.C. (2020). Harvesting Vibration Energy: Technologies and Challenges. IEEE Ind. Electron. Mag..

[4] Yang J., Zhong J.M., Chang H.L. (2018). A Closed-Loop Mode-Localized Accelerometer. IEEE/ASME J. Microelectromech. Syst..

[5] Wang J.L., Tang L.H., Zhao L.Y., Hu G.B., Song R.J., Xu K. (2020). Equivalent circuit representation of a vortex-induced vibration-based energy harvester using a semi-empirical lumped parameter approach. Int. J. Energy Res..

[6] Wu Z.X., Yang X., Wu J. (2021). Conductive Hydrogel- and Organohydrogel-Based Stretchable Sensors. ACS Appl. Mater. Interfaces.

[7] Zhao L.C., Zou H.X., Yan G., Liu F.R., Tan T., Wei K.X., Zhang W.M. (2019). Magnetic coupling and flextensional amplification mechanisms for high-robustness ambient wind energy harvesting. Energy Convers. Manag..

[8] Wu Z.X., Ding H.J., Tao K., Wei Y.M., Gui X.C., Shi W.X., Xie X., Wu J. (2021). Ultrasensitive, Stretchable, and Fast-Response Temperature Sensors Based on Hydrogel Films for Wearable Applications. ACS Appl. Mater. Interfaces.

[9] Cao H.L., Zhang Y.J., Han Z.Q., Shao X.L., Gao J.Y., Huan K., Shi Y.B., Tang J., Shen C., Liu J. (2019). Pole-zero temperature compensation circuit design and experiment for dual-mass MEMS gyroscope bandwidth expansion. IEEE-ASME Trans. Mechatron..

[10] Tao K., Yi H.P., Tang L.H., Wu J., Wang P.H., Wang N., Hu L.X., Fu Y.Q., Miao J.M., Chang H.L. (2019). Piezoelectric ZnO thin films for 2DOF MEMS vibrational energy harvesting. Surf. Coat. Technol..

[11] Shearwood C., Yates R. (1997). Development of an electromagnetic microgenerator. Electron. Lett..

[12] Meninger S., Mur-Miranda J.O., Amirtharajah R., Chandrakasan A., Lang J.H. (2001). Vibration-to-electric energy conversion. IEEE Trans. Very Large Scale Integr. Syst..

[13] Fan K.Q., Liu J., Wei D.M., Zhang D.X., Zhang Y., Tao K. (2021). A cantilever-plucked and vibration-driven rotational energy harvester with high electric outputs. Energy Convers. Manag..

[14] Wang J.L., Sun S.K., Tang L.H., Hu G.B., Liang J.L. (2021). On the use of metasurface for Vortex-Induced vibration suppression or energy harvesting. Energy Convers. Manag..

[15] Fang H.B., Liu J.Q., Xu Z.Y., Dong L., Wang L., Chen D., Cai B.C., Liu Y. (2006). Fabrication and performance of MEMS-based piezoelectric power generator for vibration energy harvesting. Microelectron. J..

[16] Wang L., Yuan F.G. (2008). Vibration energy harvesting by magnetostrictive material. Smart Mater. Struct..

[17] Tao K., Wu J., Tang L.H., Xia X., Lye S.W., Miao J.M., Hu X. (2016). A novel two-degree-of-freedom MEMS electromagnetic vibration energy harvester. J. Micromech. Microeng..

[18] Wang J.L., Hu G.B., Su Z., Li G.P., Zhao W., Tang L.H., Zhao L.Y. (2019). A cross-coupled dual-beam for multi-directional energy harvesting from vortex induced vibrations. Smart Mater. Struct..

[19] Shan X.B., Li H.L., Yang Y.C., Feng J., Wang Y.C., Xie T. (2019). Enhancing the performance of an underwater piezoelectric energy harvester based on flow-induced vibration. Energy.

[20] Wu Y., Qiu J., Zhou S., Ji H., Chen Y., Li S. (2018). A piezoelectric spring pendulum oscillator used for multidirectional and ultra-low frequency vibration energy harvesting. Appl. Energy.

[21] Chen J., Zhu G., Yang W., Jing Q., Bai P., Yang Y., Hou T.C., Wang Z.L. (2013). Harmonic-resonator-based triboelectric nanogenerator as a sustainable power source and a self-powered active vibration sensor. Adv. Mater..

[22] Lu W.L., Hwang Y.M. (2012). Analysis of a vibration-induced micro-generator with a helical micro-spring and induction coil. Microelectron. Reliab..

[23] Si H., Dong J.L., Chen L., Sun L.Z., Zhang X.D., Gao M.T. (2015). Study of the ambient vibration energy harvesting based on piezoelectric effect. Int. J. Nanosci..

[24] Huang J.K., O’Handley R.C., Bono D. New high-sensitivity hybrid magnetostrictive/electroactive magnetic field sensors. Proceedings of the Smart Structures and Materials 2003: Smart Sensor Technology and Measurement Systems.

[25] Karami M.A., Inman D.J. Nonlinear hybrid energy harvesting utilizing a piezo -magneto-elastic spring. Proceedings of the Active and Passive Smart Structures and Integrated Systems 2010.

[26] Challa V.R., Prasad M.G., Fisher F.T. (2009). A coupled piezoelectric-electromagnetic energy harvesting technique for achieving increased power output through damping matching. Smart Mater. Struct..

[27] Tao K., Chen Z.S., Yi H.P., Zhang R., Shen Q., Wu J., Tang L., Fan K., Fu Y., Miao J. (2021). Hierarchical Honeycomb-Structured Electret/Triboelectric Nanogenerator for Biomechanical and Morphing Wing Energy Harvesting. Nano-Micro Lett..

[28] Naruse Y., Matsubara N., Mabuchi K., Izumi M., Suzuki S. (2009). Electrostatic micro power generation from low-frequency vibration such as human motion. J. Micromech. Microeng..

[29] Miki D., Honzumi M., Suzuki Y., Kasagi N. Large-amplitude MEMS electret generator with nonlinear spring. Proceedings of the 2010 IEEE 23rd International Conference on Micro Electro Mechanical Systems (MEMS).

[30] Erturun U., Eisape A., West J.E. (2020). Design and analysis of a vibration energy harvester using push-pull electrostatic conversion. Smart Mater. Struct..

[31] Erturun U., Eisape A.A., Kang S.H., West J.E. (2021). Energy harvester using piezoelectric nanogenerator and electrostatic generator. Appl. Phys. Lett..

[32] Kulkarni S., Roy S., O’Donnell T., Beeby S., Tudor J. (2006). Vibration based electromagnetic micropower generator on silicon. J. Appl. Phys..

[33] Kulah H., Najafi K. (2008). Energy scavenging from low-frequency vibrations by using frequency up-conversion for wireless sensor applications. IEEE Sens. J..

[34] Wang P.H., Liu H.T., Yang Z.Q., Dai X.H., Zhao X.L. (2010). Sandwiched Electromagnetic Vibration Energy Harvester Based on MEMS Technology. Nanotechnol. Precis. Eng..

[35] Paul K., Mallick D., Roy S. (2021). Performance improvement of MEMS Electromagnetic Vibration Energy Harvester using optimized patterns of micromagnet array. IEEE Magn. Lett..

[36] Bai Y., Tofel P., Hadas Z., Smilek J., Losak P., Skarvada P., Macku R. (2018). Investigation of a cantilever structured piezoelectric energy harvester used for wearable devices with random vibration input. Mech. Syst. Signal Process..

[37] Shahruz S.M. (2006). Design of mechanical band-pass filters for energy scavenging. J. Sound Vib..

[38] Xue H.A., Hu Y.T., Wang Q.M. (2008). Broadband piezoelectric energy harvesting devices using multiple bimorphs with different operating frequencies. IEEE Trans. Ultrason. Ferroelectr. Freq. Control.

[39] Leland E.S., Wright P.K. (2006). Resonance tuning of piezoelectric vibration energy scavenging generators using compressive axial preload. Smart Mater. Struct..

[40] Li H.T., Yang Z., Zu J., Qin W.Y. (2017). Numerical and experimental study of a compressive-mode energy harvester under random excitations. Smart Mater. Struct..

[41] Khazaee M., Rezaniakolaie A., Rosendahl L. (2020). A broadband macro-fiber-composite piezoelectric energy harvester for higher energy conversion from practical wideband vibrations. Nano Energy.

[42] Liu W.Q., Liu C.Z., Ren B.Y., Zhu Q., Hu G.D., Yang W.Q. (2016). Bandwidth increasing mechanism by introducing a curve fixture to the cantilever generator. Appl. Phys. Lett..

[43] Tao K., Yi H.P., Yang Y., Tang L.H., Yang Z.S., Wu J., Chang H.L., Yuan W.Z. (2020). Miura-origami-inspired electret/triboelectric power generator for wearable energy harvesting with water-proof capability. Microsyst. Nanoeng..

[44] Zhang X.Q., Pondrom P., Sessler G.M., Ma X.C. (2018). Ferroelectret nanogenerator with large transverse piezoelectric activity. Nano Energy.

[45] Tao K., Tang L.H., Wu J., Lye S.W., Chang H.L., Miao J.M. (2018). Investigation of multimodal electret-based MEMS energy harvester with impact-induced nonlinearity. J. Microelectromech. Syst..

[46] Zhang Y.L., Wang T.Y., Luo A.X., Hu Y.S., Li X., Wang F. (2018). Micro electrostatic energy harvester with both broad bandwidth and high normalized power density. Appl. Energy.

[47] Tao K., Yi H.P., Yang Y., Chang H.L., Wu J., Tang L.H., Yang Z.S., Wang N., Hu L.X., Fu Y.Q. (2020). Origami-inspired electret-based triboelectric generator for biomechanical and ocean wave energy harvesting. Nano Energy.

[48] Tao K., Lye S.W., Tang L.H., Miao J.M., Hu X. (2015). Out-of-plane electret-based MEMS energy harvester with the combined nonlinear effect from electrostatic force and a mechanical elastic stopper. J. Micromech. Microeng..

